# A lower talar tunnel placement is associated with postoperative recurrent sprain in CLAI patients following anatomic reconstruction

**DOI:** 10.1002/jeo2.70701

**Published:** 2026-03-29

**Authors:** Haoxuan Liu, Tong Su, Yanfang Jiang, Yanbin Pi, Xing Xie, Qinwei Guo, Chen Jiao, Dong Jiang

**Affiliations:** ^1^ Department of Sports Medicine Peking University Third Hospital Beijing China; ^2^ Institute of Sports Medicine of Peking University Beijing China; ^3^ Beijing Key Laboratory of Sports Injuries Beijing China; ^4^ Engineering Research Center of Sports Trauma Treatment Technology and Devices Ministry of Education Beijing China

**Keywords:** anatomic reconstruction, anterior talofibular ligament, bone tunnel position, chronic lateral ankle instability, re‐sprain

## Abstract

**Purpose:**

To investigate whether bone tunnel position is associated with postoperative recurrent sprain in chronic lateral ankle instability (CLAI) patients following anatomic lateral ligament reconstruction with autograft and to propose potential safe zones for bone tunnel placement.

**Methods:**

Four hundred fifty‐seven CLAI patients following anatomic reconstruction at our institution from June 2015 to September 2023 were retrospectively reviewed. Among them, 30 patients sustaining recurrent sprain within 48 months after surgery were included in the recurrent sprain group. Then, 206 consecutive patients who showed no recurrent sprain with minimum 48‐month follow‐up were selected as potential controls. Patients were further divided into two groups based on whether the calcaneofibular ligament was reconstructed, and 1:1 propensity score matching was performed separately in the two groups. The positions of the fibular, talar and calcaneal tunnel were measured on three‐dimensional computed tomography (3D‐CT) scans acquired at postoperative Day 1 and were compared. Potential safe zones for tunnel placement were identified through receiver operating characteristic analysis.

**Results:**

There was no difference in fibular and calcaneal tunnel position along the reference line between groups. Patients with postoperative recurrent sprain demonstrated a significantly lower talar tunnel position on the reference line from the apex of the lateral talar process (ALTP) to the anterolateral corner of the trochlea (ACT) on the talus compared to those without postoperative recurrent sprain (58.1 ± 6.3% vs. 63.9 ± 6.0%, *p* = 0.001). The area under the curve value of the talar tunnel position was 0.744 (*p* = 0.001), and the determined cutoff value was 58.9% with a sensitivity of 90.0% and a specificity of 53.3%.

**Conclusion:**

A lower talar tunnel placement is associated with postoperative recurrent sprain in CLAI patients following anatomic reconstruction, with 58.9% above the ALTP‐ACT reference line can be a potential safe zone. The talar tunnel placement needs to be carefully considered to reduce postoperative recurrent sprain.

**Level of Evidence:**

Level III, retrospective study.

**Abbreviations**
ACT: anterolateral corner of the trochlea
AIP: anteroinferior point of the posterior subtalar joint
AITFL: anterior inferior tibiofibular ligament
ALTP: apex of the lateral talar process
AOFAS score: American Orthopaedic Foot & Ankle Society Score
AT: anterior tubercle on the fibula
ATFL: anterior talofibular ligament
AUC value: area under the curve value
BMI: body mass index
CFL: calcaneofibular ligament
CLAI: chronic lateral ankle instability
CaT: calcaneal tunnel
CaT′: the projection point of the entrance of the calcaneal tunnel
CT: computed tomography
FT: fibular tunnel
FT′: the projection point of the entrance of the fibular tunnel
ICC: intraclass correlation coefficient
IT: inferior tip
Karlsson score: Karlsson–Peterson ankle score
PP: posterior point of the posterior subtalar joint
PTFL: posterior talofibular ligament
ROC analysis: receiver operating characteristic analysis
Tegner score: Tegner Activity Level Scale
TT: talar tunnel
TT′: the projection point of the entrance of the talar tunnel
VAS score: Visual Analogue Scale

## INTRODUCTION

Lateral ankle sprain is one of the most prevalent sports injuries [34]. Due to the injuries of anterior talofibular ligament (ATFL) and calcaneofibular ligament (CFL), 20%–30% of the individuals can suffer from chronic lateral ankle instability (CLAI), which is characterized as recurrent sprain, sense of giving‐way, persistent pain, deficits of postural control and muscle strength [29]. Once rigorous conservative approaches failed, surgical intervention is considered [2]. The current gold standard surgical treatment for CLAI is the modified Broström procedure [31]. However, previous studies have demonstrated a high recurrent sprain rate following the modified Broström procedure when treating CLAI patients with risk factors such as poor ligament tissue quality, joint laxity, high athletic demands or obesity [16, 17, 23, 37].

For those patients, anatomic reconstruction with autograft or allograft is usually considered the preferred alternative to provide better stability [1, 14, 28]. The precise positioning of bone tunnels represents a critical yet challenging aspect of anatomical reconstruction techniques [10], which involves positioning the bone tunnel at the anatomical footprint of the injured ligaments. However, due to unclear or absent ligament remnants, combined avulsion fractures causing insertion site loss, morphological variations or technical constraints, surgeons may be unable to create bone tunnels at the original insertion of the ligaments, leading to non‐anatomical tunnel placement [12, 19]. However, no studies have yet investigated whether post‐operative recurrent sprain following anatomic reconstruction in CLAI cases is associated with tunnel position.

Therefore, the present study was designed to investigate whether bone tunnel position is associated with postoperative recurrent sprain recurrence in CLAI patients following anatomic reconstruction and to identify potential safe zones for bone tunnel placement based on clinical outcomes. The patients with postoperative recurrent sprain were first identified, and the propensity score matching was performed in patients without postoperative recurrent sprain to search for controls. It was hypothesized that there was significant difference of the bone tunnel placement between the patients with and without postoperative recurrent sprain. Furthermore, a cut‐off value with high classification ability could be identified. The results could make recommendations for offering more precise treatment strategies for CLAI patients following anatomic reconstruction.

## METHODS

### Patient enrolment

This was a single‐centre retrospective cohort study approved by our institutional review board. The inclusion criteria for CLAI patients underwent anatomic reconstruction using an autograft [35]: (1) a traumatic history of at least one significant ankle sprain, and the most recent ankle sprain occurred >3 months prior to study participation; (2) a history of the previously injured ankle joint ‘giving way’, and/or recurrent sprain and/or ‘feelings of instability’; (3) Grade III chronic ATFL and CFL injury confirmed by magnetic resonance imaging; (4) positive anterior drawer test with increased displacement of more than 3 mm compared to the contralateral side; (5) failure of at least of 6 months of rigorous conservative treatment and the decision to undergo surgery (6) arthroscopic exploration using a probe confirmed the ATFL and/or CFL had poor tension or was missing that could not be repaired and underwent anatomic reconstruction of ATFL and/or CFL using an autograft.

Patients were excluded if they had: (1) other ankle ligament injury, ankle fracture or bilateral ankle instability; (2) central and/or peripheral neuromuscular disease; (3) progressing ankle arthritis (Takakura stage ≥ 3); or (4) lost to the final follow‐up or unwilling to engage in the study.

All follow‐up and clinical records of 457 patients who underwent ligament reconstruction at our institution from June 2015 to September 2023 were retrospectively reviewed to identify patients who experienced recurrent sprain within 48 months postoperatively. Recurrent sprain was defined as a non‐contact injury (i.e., an injury occurring without direct physical contact, collision or external force, resulting from sudden unbalanced movements such as landing, twisting or cutting under the individual's own body weight and muscle contraction) presenting as inversion trauma with lateral ankle pain requiring at least 1 day to return to daily activities.

**Figure 1:**
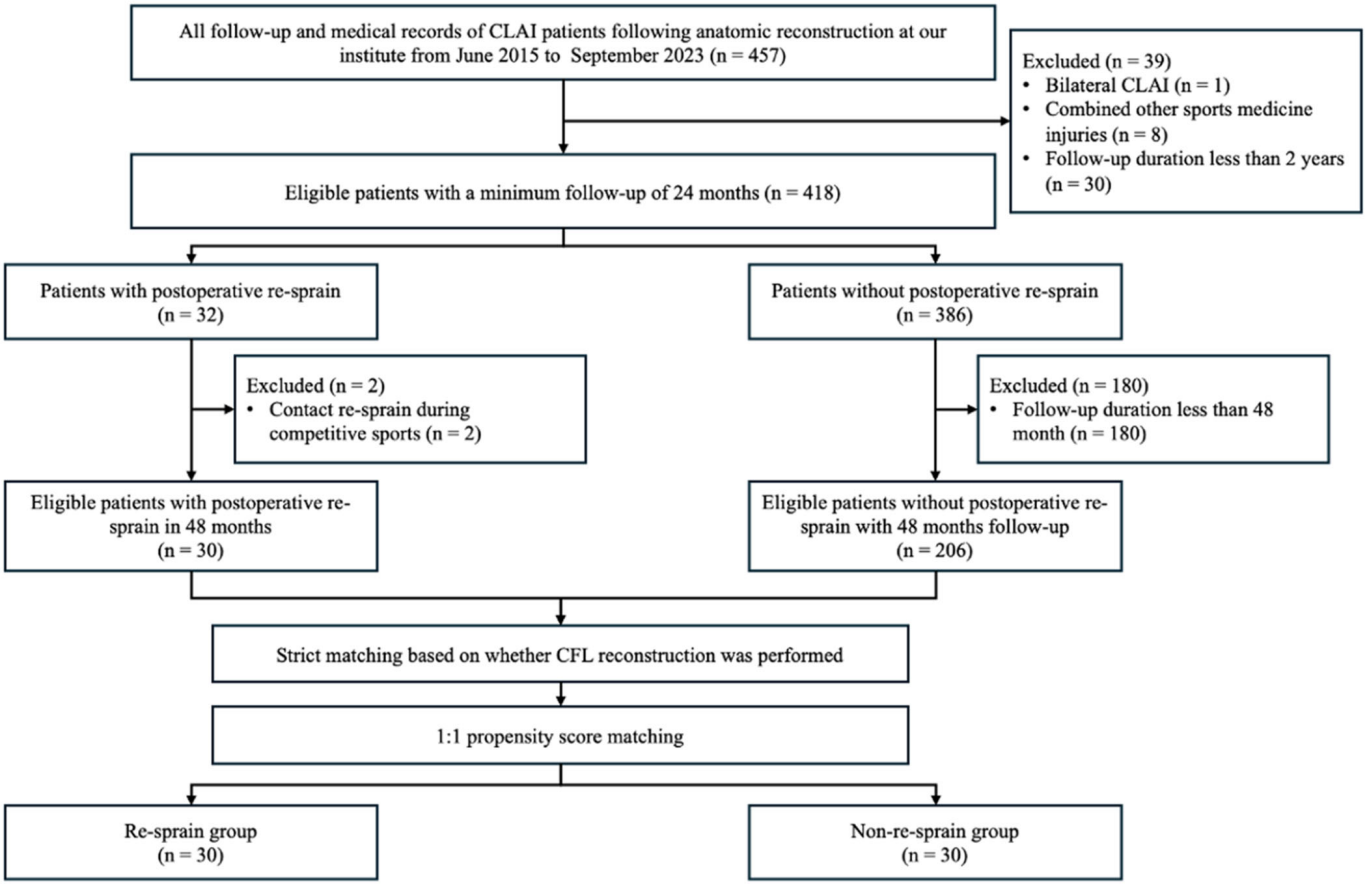
Patient enrolment. CFL, calcaneofibular ligament; CLAI, chronic lateral ankle instability.

Then, 206 consecutive eligible patients who reported no postoperative recurrent sprain at the 48‐month follow‐up were selected as potential controls. Since the reconstruction of the CFL or not influences the placement of fibular tunnel (FT) [27], the 236 patients were initially divided into two groups based on whether CFL reconstruction was performed. Then, to minimize confounding of other potential factors, a 1:1 propensity score matching was conducted within the two groups separately based on age at surgery, gender, body mass index (BMI), post‐injury duration, pre‐injury Tegner score, the presence of os subfibulare, the presence of osteochondral lesion of the talus and follow‐up duration. As shown in Figure 1, according to the matching results, 30 patients without postoperative recurrent sprain were included in the non‐recurrent sprain group.

### Surgical procedure

All anatomic reconstructions using an autograft for CLAI patients were performed according to previous studies [26, 27]. Patients underwent either spinal or general anaesthesia and were placed in a supine position with the ipsilateral hip elevated approximately 30°. Standard anteromedial and anterolateral portals were established for diagnostic arthroscopy using a 2.9 mm 30° arthroscopic system (Smith & Nephew) to assess and manage intra‐articular pathologies, including osteochondral lesions, osteophytes and loose bodies. The ipsilateral gracilis tendon was then harvested and folded. A 4–5 cm oblique incision was made anteroinferior to the fibula. When the CFL remnant exhibited excellent quality or remained intact, it was either repaired or left untreated. Following debridement of the ATFL footprints, two bone tunnels were drilled for isolated ATFL reconstruction using the prepared tendon. If the CFL remnant quality was poor, a Y‐shaped tunnel configuration was created: an FT was drilled at the common insertion centre of the ATFL and CFL on the fibula, while talar and calcaneal tunnels were drilled at the respective insertion centres of the ATFL and CFL, enabling anatomical reconstruction of both ligaments with the harvested tendon. When the remnant of ATFL and/or CFL was absent, the tunnel was drilled through the surgeons' estimation of the anatomic footprints of the ligaments based on anatomical landmarks, including fibular obscure tubercle (FOT) for FT and apex of the lateral talar process (ALTP) for talar tunnel (TT), combined with preoperative computed tomography (CT) and magnetic resonance imaging (MRI). The tendon was then introduced into the tunnels with the help of a guide pin and fixed by PEEK (polyether ether ketone) interference screws (5 mm Milagro), respectively, in neutral ankle position.

### Postoperative rehabilitation

After the surgery, the ankle was immobilized with short leg cast for the first 2 weeks. Partial weight‐bearing was allowed at Week 3 postoperatively and gradually transitioned to full weight‐bearing with a walking brace. From Week 6, weight‐bearing muscle strength exercise was allowed, and dynamic balance exercise started at Week 7. All the patients returned to full weight‐bearing walk and sport based on their tolerance. Then, patients were then able to resume their daily activities and engage in sports that fell within their physical capabilities.

### Three‐dimensional CT (3D‐CT) evaluation

**Figure 2:**
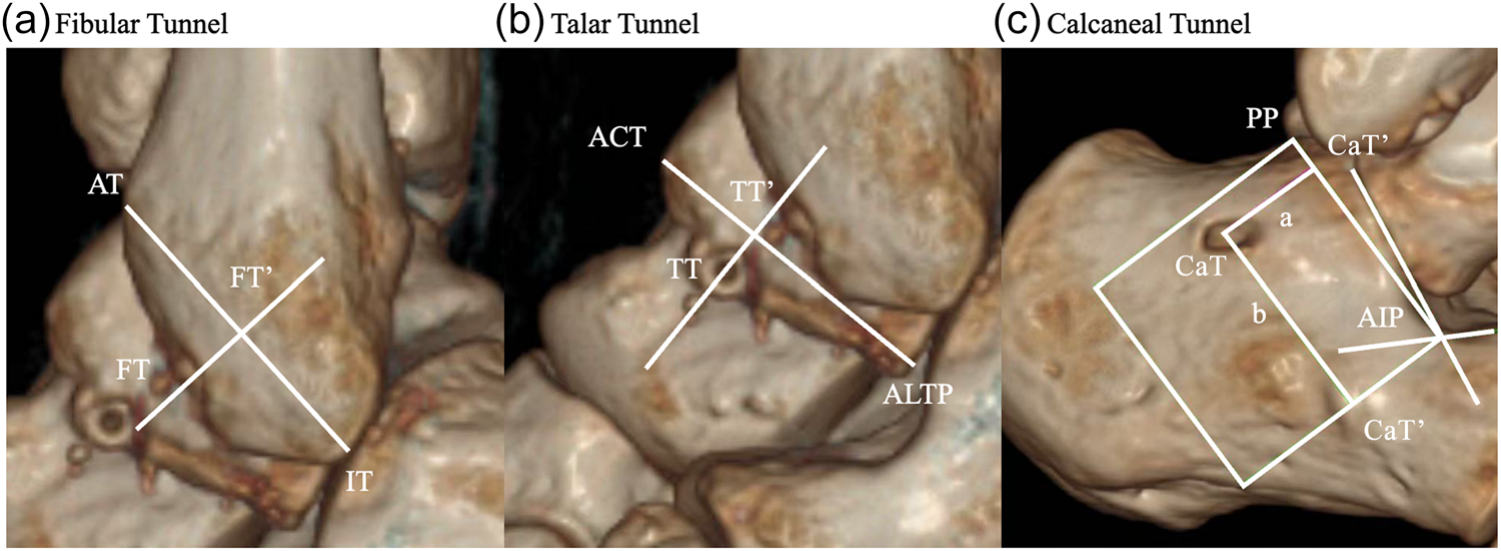
3D‐CT measurement methods of the (a) fibular, (b) talar and (c) calcaneal tunnel position. 3D‐CT, three‐dimensional computed tomography; ACT, anterolateral corner of the trochlea; AIP, anteroinferior point; ALTP, apex of the lateral talar process; AT, anterior tubercle of the fibula; CaT, calcaneal tunnel; CaT′, the projection point of the entrance of the calcaneal tunnel; FT, fibular tunnel; FT′, the projection point of the entrance of the fibular tunnel; IT, inferior tip of the fibula; PP, posterior point of the posterior subtalar joint; TT, talar tunnel; TT′, projection point of the entrance of the talar tunnel.

A multichannel CT scanner (uCT 790; United Imaging) was used to acquire CT scans at Day 1 postoperatively. The ankle was immobilized by a cast in the neutral position. The scan parameters included a 512 × 512 matrix, 0.5‐mm slice thickness, 8 s scan time, 120 kV and 100 mA. All images were reformatted (including multiple planes reformat and volume rendering), and measurements were conducted by AW Server 4.7 (GE Healthcare). As shown in Figure 2, according to previous studies, the anatomic footprints of the reference line were first identified [4, 18, 32], and percentage measurement was used to minimize the topographical impact of the fibula and the talus [13, 15].

For the FT, the distance from the inferior tip of the fibula (IT) to the anterior tubercle of the fibula (AT) was first measured. The centre of the FT entrance was projected orthogonally onto the AT‑IT reference line, and the intersection was defined as FT′. The distance from FT′ to AT was measured, and its relative position was calculated as a percentage of the total AT‑IT length. For the TT, the distance from the ALTP to the anterolateral corner of the talar trochlea (ACT) was measured. The centre of the TT entrance was projected orthogonally onto the ALTP‑ACT reference line, with the intersection defined as TT′. The distance from TT′ to ALTP was measured, and its relative position was expressed as a percentage of the total ALTP‑ACT length. For the calcaneal tunnel (CaT), the anteroinferior point (AIP) and posterior point (PP) of the posterior subtalar joint were identified. A reference line connecting AIP and PP was established. The entrance of the CaT was projected orthogonally onto this reference line. Its position was determined by two distances: projection Distance a (along the orthogonal direction) and Distance b (along the AIP‑PP direction).

The inter‐observer reliability and intra‐observer reproducibility of the measurement methods were first examined according to previous studies [3, 12]. To evaluate the inter‐observer reliability of the evaluation methods, two authors performed measurements on the 3D‐CT images of all patients. To assess the intra‐observer reproducibility of the evaluation methods, the measurements were repeated initially and after a 2‐week interval. The intraclass correlation coefficient (ICC) of the inter‐observer reliability and the intra‐observer reproducibility was then calculated. After the ICC tests, the order of the selected patient list was randomized to mitigate potential bias, and one author completed all measurements to reduce inter‐observer variability.

### Clinical evaluation

Patient demographics, including age at surgery, gender, height, weight, BMI, post‐injury duration (the length of time elapsed between the first occurrence of the significant ankle sprain and the date of surgical intervention) and pre‐injury Tegner score were collected from preoperative medical records. Intraoperative findings such as calcaneofibular ligament reconstruction, the presence of os subfibulare, the presence of osteochondral lesion of the talus and the presence of osteophyte were collected from surgical records.

### Statistical analysis

PASS Sample Size Software 15 (NCSS, LLC) was used for sample size calculation. Since no previous studies have compared the bone tunnel position of the patients with and without postoperative recurrent sprain, the power of the study was calculated based on the effect size (0.907 for ALTP‐TT′ and 0.928 for TT′ position) of the evaluation methods with examined significant differences. When an α was set at 0.05, the study achieved an actual power of 0.932 for ALTP‐TT′ and 0.942 for TT′ position.

All the data were analysed by IBM SPSS Statistics 24.0 (SPSS Inc.). The Shapiro–Wilk test was first performed to check for normal distribution of each group. The data were presented as the mean ± standard deviation for continuous variable, and frequency (percentage) for categorical data. The Pearson's *χ* ^2^ test, Mann–Whitney *U* test or independent *t* test (two‐sided test and *α* = 0.05) was applied to compare the differences between groups. Differences were considered to be significant at *p* < 0.05.

The ICC was used to evaluate the test‐retest repeatability of the evaluation methods, which could be rated as excellent (ICC > 0.9), good (0.75 < ICC < 0.9), moderate (0.5 < ICC < 0.75) or poor (ICC < 0.5) [3].

The receiver operating characteristic (ROC) analysis was performed to evaluate the predictive ability based on the area under the curve (AUC) and to determine the optimal cutoff value according to the maximum Youden index [25].

## RESULTS

### Patient demographics

As shown in Table 1, there were no significant differences in all patient demographics between the patient with and without postoperative recurrent sprain.

**Table 1:** Comparison of the patient demographics of the patients with and without postoperative recurrent sprain.

	Recurrent sprain (yes, *n* = 30)	Recurrent sprain (no, *n* = 30)	*p* value
Gender, male/female (%)	18 (60.0)/12 (40.0)	17 (56.7)/13 (43.3)	0.793
Side, right/left (%)	17 (56.7)/13 (43.3)	18 (60.0)/12 (40.0)	0.793
Age at surgery, years	25.93 ± 7.59	27.77 ± 7.11	0.338
Height, cm	172.37 ± 8.53	172.20 ± 9.17	0.942
Weight, kg	70.70 ± 10.60	72.17 ± 12.99	0.605
BMI, kg/m^2^	23.75 ± 2.85	24.21 ± 2.95	0.542
Post‐injury duration, months	38.30 ± 49.33	43.07 ± 45.89	0.796
Pre‐injury Tegner score	5.93 ± 0.74	6.33 ± 0.99	0.267
CFL reconstruction, yes/no (%)	12 (40.0)/18 (60.0)	12 (40.0)/18 (60.0)	1.000
Os subfibulare, yes/no (%)	10 (33.4)/20 (66.7)	8 (26.7)/22 (73.3)	0.573
Osteochondral lesion of the talus, yes/no (%)	11 (36.7)/19 (63.3)	12 (40.0)/18 (60.0)	0.791
Osteophyte, yes/no (%)	9 (30.0)/21 (70.0)	11 (36.7)/19 (63.3)	0.584

Abbreviations: BMI, body mass index; CFL, calcaneofibular ligament.

### Comparison of the bone tunnel position of the patients with and without postoperative recurrent sprain

The inter‐observer reliability and intra‐observer reproducibility for the included measurement methods were excellent, with all ICCs > 0.900, as shown in Table 2.

**Table 2:** ICC test of the inter‐observer agreement and intra‐observer agreement.

	Inter‐observer reliability		Intra‐observer reproducibility	
	ICC (95% CI)	*p* value	ICC (95% CI)	*p* value
ATFL (*n* = 60)				
FT′ position	0.969 (0.948–0.981)	<0.001*	0.988 (0.980–0.993)	<0.001*
TT′ position	0.967 (0.945–0.980)	<0.001*	0.990 (0.984–0.994)	<0.001*
CFL (*n* = 24)				
CaT′ position (orthogonal direction)	0.967 (0.889–0.990)	<0.001*	0.979 (0.929–0.994)	<0.001*
CaT′ position (AIP‐PP direction)	0.963 (0.879–0.989)	<0.001*	0.976 (0.918–0.993)	<0.001*

Abbreviations: AIP, anteroinferior point of the posterior subtalar joint; ATFL, anterior talofibular ligament; CaT′, the projection point of the entrance of the calcaneal tunnel; CFL, calcaneofibular ligament; CI, confidence interval; FT′, the projection point of the entrance of the fibular tunnel; ICC, intraclass correlation coefficient; PP, posterior point of the posterior subtalar joint; TT′, the projection point of the entrance of the talar tunnel.

* Significant difference between groups.

As shown in Table 3, there were no significant differences in the lengths of the IT‐AT reference line, the ALTP‐ACT reference line and the AIP‐PP reference line between the two groups. Additionally, there were no significant differences in the distance from the projected position of fibular tunnel and calcaneal tunnel to the reference position or on the reference line between groups. This indicates that the positions of the fibular and calcaneal bone tunnels are relatively consistent between groups. However, there is a significant difference in the position of the talar bone tunnels between the two groups of patients. Compared to the patients without postoperative recurrent sprain, the patients with postoperative recurrent sprain demonstrated a significantly shorter distance between ALTP and TT′ (16.6 ± 2.7 vs. 19.0 ± 2.6, *p* = 0.001), and significantly lower TT and TT′ positions (58.1 ± 6.3 vs. 63.9 ± 6.0, *p* = 0.001).

**Table 3:** Comparison of the bone tunnel position of the patients with and without postoperative recurrent sprain.

	Recurrent sprain (yes, *n* = 30)	Recurrent sprain (no, *n* = 30)	*p* value
Fibular side			
IT‐AT (mm)	27.2 ± 3.9	26.1 ± 5.3	0.385
FT′‐IT (mm)	10.8 ± 2.9	11.0 ± 2.7	0.792
FT′ position (%)	42.7 ± 6.6	44.2 ± 5.8	0.347
Talar side			
ALTP‐ACT (mm)	28.6 ± 3.4	29.8 ± 3.4	0.196
TT′‐ALTP (mm)	16.6 ± 2.7	19.0 ± 2.6	0.001*
TT′ position (%)	58.1 ± 6.3	63.9 ± 6.0	0.001*

	Recurrent sprain (yes, *n* = 12)	Recurrent sprain (no, *n* = 12)	*p* value
Calcaneal side			
AIP‐PP (mm)	29.7 ± 2.5	30.5 ± 1.6	0.358
Distance a (mm)	12.7 ± 1.8	13.3 ± 2.2	0.456
Distance b (mm)	23.2 ± 3.4	24.0 ± 1.9	1.000
CaT′ position (orthogonal direction)	42.9 ± 6.4	43.7 ± 7.4	0.777
CaT′ position (AIP‐PP direction)	77.9 ± 9.9	78.8 ± 5.9	0.684

Abbreviations: ACT, anterolateral corner of the trochlea; AIP, anteroinferior point of the posterior subtalar joint; ALTP, apex of the lateral talar process; AT, anterior tubercle on the fibula; CaT′, the projection point of the entrance of the calcaneal tunnel; FT′, the projection point of the entrance of the fibular tunnel; IT, inferior tip of the fibula; PP, posterior point of the posterior subtalar joint; TT′, the projection point of the entrance of the talar tunnel.

* Significant difference between groups.

### The classification ability and the cutoff value of the evaluation methods

**Figure 3:**
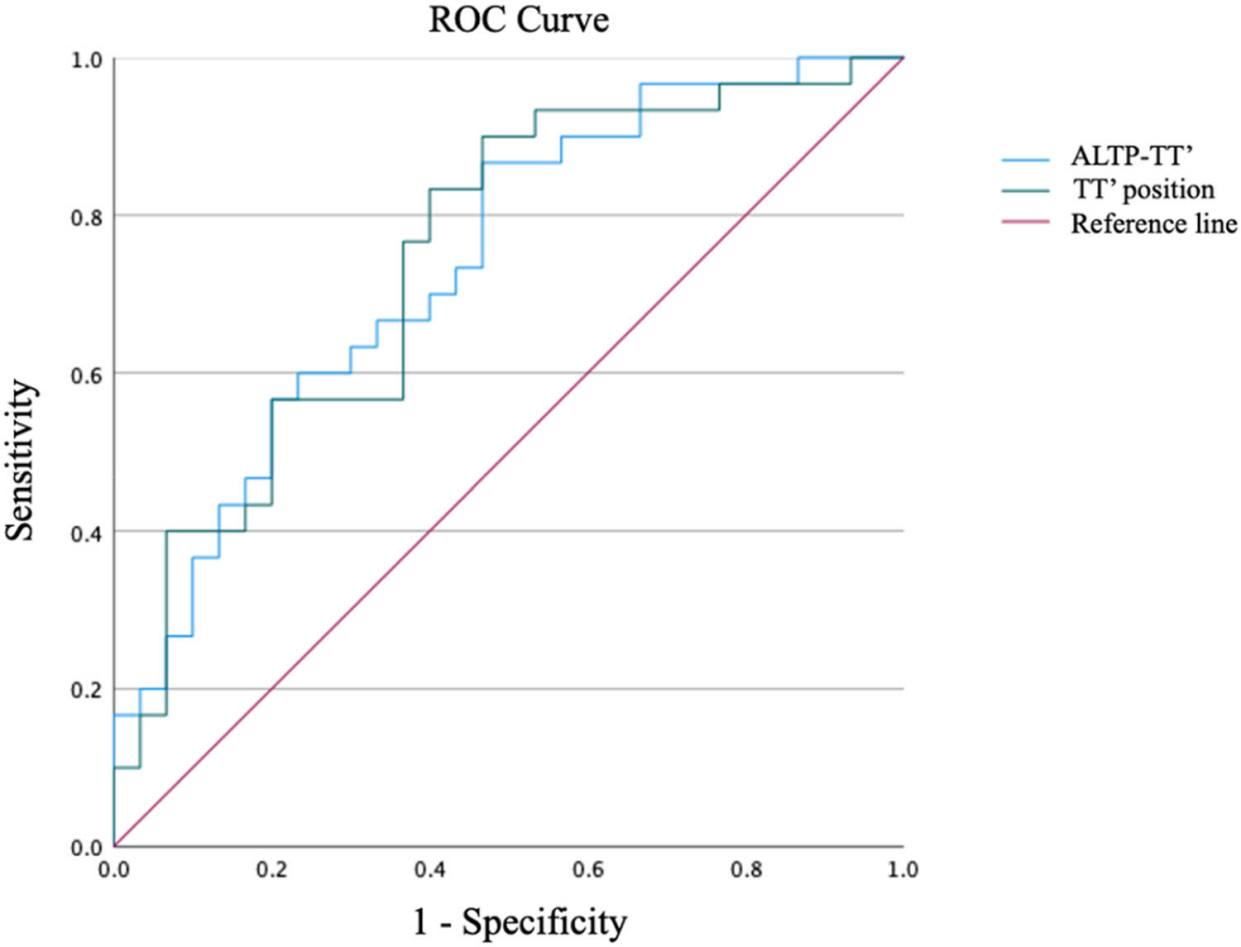
The ROC curve of the radiographic evaluation methods. ALTP, apex of the lateral talar process; ROC, receiver operating characteristic; TT′, the projection point of the entrance of the fibular tunnel.

As shown in Table 4 and Figure 3, ROC analysis demonstrated that using TT′ to describe the position of the talar tunnel provided the highest AUC value of 0.744 (95% confidence interval [CI] 0.619–0.870). The cutoff value determined by the Youden index was 16.4 mm for the ALTP‐TT′ distance and 58.9% for the TT′ position.

**Table 4:** The classification ability and the cutoff value of the evaluation methods.

	AUC (95% CI)	*p* value	Cut‐off point	Sensitivity	Specificity
ALTP‐TT′	0.739 (0.614–0.863)	0.001*	16.4 mm	86.7%	53.3%
TT′ position	0.744 (0.619–0.870)	0.001*	58.9%	90.0%	53.3%

Abbreviations: AUC, area under the curve; ALTP, apex of the lateral talar process; CI, confidence interval; TT′, the projection point of the entrance of the talar tunnel.

* Significant difference between groups.

## DISCUSSION

The main finding of our study was that for patients following anatomic reconstruction using an autograft, the talar tunnel placement in patients with postoperative recurrent sprain was lower than that in patients without postoperative recurrent sprain. The result of the ROC curve analysis suggests that the talar tunnel placement should be above the 58.9% of the ALTP‐ACT line. Therefore, the talar tunnel placement needs to be carefully considered preoperatively and intraoperatively to reduce postoperative recurrent sprain.

In our study, there was no significant difference in the projection point of the entrance of the fibular tunnel positions between the two groups. This suggests that the current reconstruction techniques can effectively place the fibular tunnel within the anatomic position. The high rate of anatomically positioned fibular tunnels may be attributed to the comprehensive exploration of the native fibular insertion of the ATFL and the common fibular insertion of the ATFL and CFL [4, 7, 8, 22, 30]. Besides, various reliable anatomical landmarks that can be easily identified during the surgery also contribute to accurate positioning of the fibular tunnel. The most widely utilized landmark is the FOT [11, 18]. As a structure easily identifiable under both open and arthroscopic visualization [9, 36], it largely prevents non‐anatomical placement of the fibular tunnel. Besides, as suggested by previous studies, the fibular insertion of the posterior talofibular ligament (PTFL) and the distal fascicle of the anterior inferior tibiofibular ligament (AITFL) can additionally serve as a guide for fibular tunnel placement [6, 21, 33]. These intraoperatively discernible anatomical landmarks facilitate relatively straightforward placement of the fibular tunnel in or near its anatomical position.

In patients in the non‐recurrent sprain group, the projection point of the talar tunnel entrance was located at an average of 63.9% along the ALTP‐ACT line. This value aligns closely with the anatomical centres of the ATFL's talar and fibular insertions reported in previous anatomical studies. In the anatomical study by Clanton et al., the distance from the talar insertion of the superior bundle of the ATFL to ALTP was 65.7% of the total ALTP‐ACT distance, which ranges from 64.1% to 67.3% [4]. Considering that they used direct distance ratios rather than orthogonal projections onto the ALTP‐ACT line, the theoretical anatomical position—after accounting for the influence of vertical distance—would likely be similar to the findings in our study. A recent anatomical study by Wang et al. found that after reconstructing the ATFL in cadaveric models, the projection point of the entrance centre of the reconstructed talar tunnel was located at 63.5% of the ALTP‐ACT distance, which ranges from 60.8% to 66.2% on the 3D‐CT measurements [32]. These studies collectively indicate that the centre of the ATFL's talar insertion is generally located at the talar body–neck junction, approximately from 60% to 68% along the ALTP‐ACT line [19], which is consistent with previous clinical studies [15].

**Figure 4:**
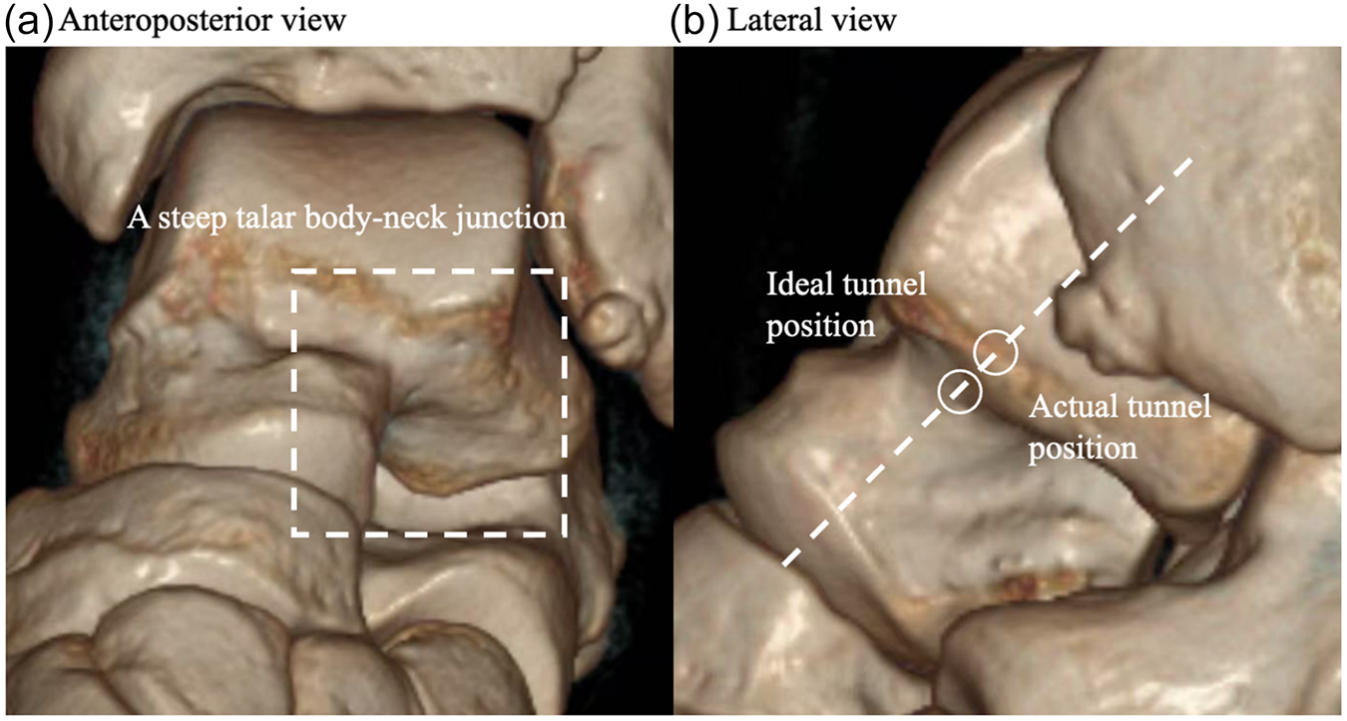
The (a) anteroposterior and (b) lateral 3D‐CT views of a CLAI patient with steep talar body–neck junction. Under this circumstance, drilling at the ideal tunnel position is relatively unstable and might lead to a short talar tunnel. Therefore, the talar tunnel was after a horizontal posterior displacement of the tunnel along the long axis of the talus onto the talar body, which resulted in a lowered position on the ALTP‐ACT line. 3D‐CT, three‐dimensional computed tomography; ACT, anterolateral corner of the trochlea; ALTP, apex of the lateral talar process; CLAI, chronic lateral ankle instability.

However, the CLAI patients with postoperative recurrent sprain exhibited lower talar tunnel position compared to those without postoperative recurrent sprain. ROC curve analysis indicated that the talar tunnel should be positioned above 58.9% of the total length of the ALTP‐ACT reference line. This phenomenon appears to have been similarly observed in the cadaveric study by Michels et al. [20]. In their study, 2 of the 14 reconstructed talar tunnels demonstrated a positional deviation greater than 2 mm from the original insertion site, whereas all 14 fibular tunnels were located within 2 mm of the ligament insertion, suggesting that the talar tunnel is more likely to be positioned non‐anatomically compared to the fibular tunnel, which is consistent with our results. Therefore, the talar tunnel placement needs to be carefully considered preoperatively and intraoperatively to reduce postoperative recurrent sprain. To further investigate the causes of non‐anatomically positioned tunnels, we retrospectively reviewed the surgical documentation of the 16 patients in the recurrent sprain group with tunnel position below 58.9% of the reference line. It was found that 6 of 16 patients have been reported as complete absence of the ATFL. In such cases, the original insertion of the ATFL cannot be identified. Although the ALTP, ACT and talar obscure tubercle have all been reported in previous studies as potential references for talar tunnel placement [5, 8, 18, 24], previous studies also acknowledge the shortage that ALTP and ACT cannot be easily exposed during the surgery unless a large incision was made [15], while the talar obscure tubercle can only be identified in 58% of the patients [18]. This highlights the need for further anatomical studies to identify potential anatomical landmarks for talar tunnel placement. Additionally, we also retrospectively reviewed the preoperative and postoperative 3D‐CT scans of the patients, and 6 of the remaining 10 patients exhibited an excessively steep talar body‐neck junction, revealing another cause of non‐anatomical tunnel placement (Figure 4). In such cases, drilling the talar tunnel at the original insertion of the ATFL was not feasible, so the talar tunnel was positioned after a horizontal posterior displacement of the tunnel along the long axis of the talus onto the talar body, which resulted in a downward shift along the ALTP‐ACT line, lowering its position on the ALTP‐ACT line. However, further biomechanical studies are necessary to further investigate this phenomenon. Also, it should be noted that although our analysis found the difference of the mean tunnel positions between the two groups was only 5.8%, the cut‐off value identified by ROC analysis demonstrated a sensitivity of 90%, indicating that this finding holds significant clinical value. However, the specificity of our study was only 53.3%, suggesting that there are other factors contributing to recurrent sprains after ligament reconstruction in patients, which require further investigation.

To our knowledge, this is the first study to investigate the impact of tunnel position on postoperative recurrent sprain following anatomic reconstruction in patients with CLAI. The results of the study indicate that a lower talar tunnel position along the ALTP‐ACT reference line is associated with postoperative recurrent sprain, and 58.9% of the ALTP‐ACT reference line may serve as a safe threshold for the talar tunnel placement. Given the previously reported recurrence rates of 5%–7% at the 24–48 months' follow‐up after anatomic reconstruction, approximately 12% of talar tunnels may be placed below the anatomical safe threshold due to factors such as absent or indistinct ligament remnant, morphological variations and intraoperative technical issues, which was also partially consistent with the study by Michels et al. [20]. Therefore, the potential safe zone identified in our study can serve as an auxiliary reference in preoperative and intraoperative planning to help avoid non‐anatomical tunnel placement in those cases. However, we must acknowledge that the 1:1 propensity score matching design somehow limits the generalizability of our conclusions. Since the overall recurrent sprain rate after ligament reconstruction remains relatively low, the safe zone identified in the present study might still deviate from the actual safe threshold. Nevertheless, the cutoff value identified in the present study was consistent with the lower limit of anatomical position of the original insertion of the ligament reported in previous anatomical studies, suggesting its clinical relevance.

There are some limitations to this study. First, all analyses were conducted post hoc based on clinical outcomes, which prevented our study from investigating the potential impact of excessively high tunnel placement on postoperative recurrent sprain. Additionally, since only 12 of the patients in each group combined CFL reconstruction, it is hard to determine whether the position of CFL tunnel is associated with postoperative recurrent sprain due to the limited sample sizes. Therefore, comprehensive studies with larger sample size are required to further investigate the influence of non‐anatomically positioned bone tunnel on CLAI patients following anatomic reconstruction.

## CONCLUSION

A lower talar tunnel placement is associated with postoperative recurrent sprain in CLAI patients following anatomic reconstruction, with 58.9% above the ALTP‐ACT reference line can be a potential safe zone. The talar tunnel placement needs to be carefully considered to reduce postoperative recurrent sprain.

## AUTHOR CONTRIBUTIONS

All authors contributed to the study conception and design. Conception and design of the study was performed by Dong Jiang. Acquisition of data was performed by Haoxuan Liu and Tong Su. Analyses of data were performed by Yanbing Pi, Xing Xie and Qinwei Guo. Drafting the work was performed by Haoxuan Liu, Xing Xie and Yangfang Jiang. Revising it critically for important intellectual content was performed by Chen Jiao and Dong Jiang. Final approval of the version to be published was performed by Haoxuan Liu, Tong Su, Yanfang Jiang, Yanbin Pi, Xing Xie, Qinwei Guo, Chen Jiao and Dong Jiang.

## CONFLICT OF INTEREST STATEMENT

The authors declare no conflicts of interest.

## ETHICS STATEMENT

This study received approval from the IRB Medical Committee of Peking University Third Hospital, IRB00006761‐M20250961. The authors affirm that all participants provided informed consent for publication.

## Data Availability

The datasets used and/or analysed during the current study are available from the corresponding author on reasonable request.
